# More skilled clinical management of COVID-19 patients modified mortality in an intermediate respiratory intensive care unit in Italy

**DOI:** 10.1186/s12931-021-01613-2

**Published:** 2021-01-15

**Authors:** Giovanna E. Carpagnano, Giovanni Migliore, Salvatore Grasso, Vito Procacci, Emanuela Resta, Francesco Panza, Onofrio Resta

**Affiliations:** 1 Department of Basic Medical Science, Institute of Respiratory Disease, Neuroscience, and Sense Organs, University of Bari “Aldo Moro”, Bari, Italy; 2 General Direction, Policlinico Hospital, Bari, Italy; 3 Department of Emergency and Organ Transplantation, Section of Anesthesia and Intensive Care, University of Bari “Aldo Moro”, Bari, Italy; 4 Emergency Department, Policlinico Hospital, Bari, Italy; 5 Translational Medicine and Health System Management, University of Foggia, Foggia, Italy; 6 Population Health Unit, Healthy Aging Phenotypes Research Unit, “Salus in Apulia Study”, National Institute of Gastroenterology “Saverio de Bellis”, Research Hospital, Castellana Grotte, Bari, Italy

**Keywords:** Critical care, Assisted ventilation, Intermediate RICU, Survival, Pandemic, Italy

## Abstract

**Background:**

Some studies investigated epidemiological and clinical features of laboratory-confirmed patients with severe acute respiratory syndrome coronavirus 2 (SARS-CoV-2) the virus causing coronavirus disease 2019 (COVID-19), but limited attention has been paid to the follow-up of hospitalized patients on the basis of clinical setting and the expertise of clinical management.

**Methods:**

In the present single-centered, retrospective, observational study, we reported findings from 87 consecutive laboratory-confirmed COVID-19 patients with moderate-to-severe acute respiratory syndrome hospitalized in an intermediate Respiratory Intensive Care Unit (RICU), subdividing the patients in two groups according to the admission date (before and after March 29, 2020).

**Results:**

With improved skills in the clinical management of COVID-19, we observed a significant lower mortality in the T2 group compared with the T1 group and a significantly difference in terms of mortality among the patients transferred in Intensive Care Unit (ICU) from our intermediate RICU (100% in T1 group vs. 33.3% in T2 group). The average length of stay in intermediate RICU of ICU-transferred patients who survived in T1 and T2 was significantly longer than those who died (who died 3.3 ± 2.8 days *vs*. who survived 6.4 ± 3.3 days). T

**Conclusions:**

The present findings suggested that an intermediate level of hospital care may have the potential to modify survival in COVID-19 patients, particularly in the present phase of a more skilled clinical management of the pandemic.

## Background

The need to determine the full spectrum and natural history of the pandemic of severe acute respiratory syndrome coronavirus 2 (SARS-CoV-2), the virus causing coronavirus disease 2019 (COVID-19), is to inform clinical management and public health decision making. In Italy, the outbreak of COVID-19 officially started on 30 January 2020, and in less than two weeks, the number of cases increased beyond expectations, putting the Italian health service under considerable strain [1]. On 11 March 2020, the World Health Organization (WHO) declared the current COVID-19 outbreak a pandemic, with the outbreak resulting in more than 21 million cases and over 760,000 deaths worldwide as of 16 August 2020 [2, 3]. COVID-19 patients develop acute respiratory distress syndrome (ARDS), require respiratory support [4], and may require hospitalization in Intensive Care Unit (ICU). Defined as Type 1 respiratory failure, there is hypoxia (PaO2 < 8 kPa), without hypercapnia (carbon dioxide retention or PaCO2), with patients commonly presenting with hypoxia worsening, and additional signs such as tachypnoea, increased use of accessory muscles, tachycardia, pale and cold peripheries, sweating, confusion, agitation or reduced level of consciousness and cyanosis [5]. In China, the percentage of COVID-19 patients who required ICU hospitalization varied from 5 to 32% [6]. However, findings from China and Italy suggested high mortality and stressed ICU capacity of care [7, 8]. Recently, Siddiqi and Mehra proposed a staged progression model based on observed clinical courses in published studies [9]. This 3-stage clinical classification system suggested that COVID-19 illness may exhibit 3 grades of increasing severity, which correspond with distinct clinical findings, response to therapy, and clinical outcome [9]. In Stage I, or the mild phase, the virus multiplies and establishes residence in the host, predominantly in the respiratory tract. In Stage II, or the moderate phase, there is viral multiplication and localized inflammation in the lungs without (Stage IIa) and with hypoxia (Stage IIb). Stage III is marked by extra-pulmonary systemic hyperinflammation syndrome [9]. The prognosis and recovery from Stage 3 is generally poor. Rapid recognition of which stage the patient is and the development of appropriate therapy may have the greatest yield [9].

Although previous studies focused on clinical management with non-invasive respiratory support of COVID-19 patients in ICU [6–8], there is no evidence coming from an intermediate Respiratory Intensive Care Unit (RICU) or noninvasive respiratory care unit [8], a model of care designed for monitoring and treating respiratory patients whose illness is at a level of severity that is intermediate between that which requires ICU facilities and that which can be managed on a conventional ward. An intermediate RICU is an area for monitoring and treating patients with acute or exacerbated respiratory failure caused by a disease that is primarily respiratory [10, 11]. While some studies investigated epidemiological and clinical features of laboratory-confirmed COVID-19 patients [6, 12], limited attention has been paid to the follow-up of hospitalized patients on the basis of clinical setting and the expertise of clinical management.

## Methods

In the present single-centered, retrospective, observational study, we reported findings from 87 consecutive laboratory-confirmed COVID-19 patients, all with moderate-to-severe ARDS, hospitalized in an intermediate RICU [10, 11], Policlinico University Hospital, Bari, Italy and collected from March 11 to April 17, 2020. The present study adhered to the Strengthening the Reporting of Observational Studies in Epidemiology” (STROBE) guidelines (https://www.strobe-statement.org/index.php?id=strobe-home), the “Standards for Reporting Diagnostic Accuracy Studies” (STARD) guidelines (http://www.stard-statement.org/), and was conducted in accordance with the Helsinki Declaration of 1975. After initial screening at the Emergency Department, the patients were admitted to our intermediate RICU or in an ICU accordingly to the illness severity. The present study was approved by the Policlinico Hospital of University of Bari “Aldo Moro” institutional review board and informed consent was obtained from all subjects involved in the present analyses.

Laboratory-confirmed COVID-19 patients were affected by ARDS defined according to the Berlin definition, so a respiratory failure characterized by arterial oxygen partial pressure to fractional inspired oxygen ratio (PaO2/FiO_2_) < 300 mmHg despite PEEP > 5 cmH_2_O, associated to bilateral chest opacities (not fully explained by effusions, lobar/lung collapse or nodules) with an acute onset, within 1 week of a known clinical insult or new or worsening respiratory symptoms [13]. In these laboratory-confirmed COVID-19 patients, apart from moderate to severe hypercapnic patients, who clearly needed the bilevel positive airway pressure (BPAP) respiratory support rather than continuous positive airway pressure (CPAP) respiratory support, our choice was driven by patient’s clinical evaluation. After a CPAP trial with a progressive pressure raising up to 12–15 cmH_2_O (when needed), if respiratory rate still > 30 we decided to switch CPAP to BPAP. High respiratory rate in ARDS patients is an indicator of respiratory fatigue, and BPAP can reduce work of breathing giving relief in these patients [14]. Laboratory-confirmed COVID-19 patients affected by severe ARDS, non responding to NIV, in which intubation and ICU transfer would not modify their outcome according to resuscitator counseling, remained in our intermediate RICU. Therefore, all patients who did not respond to NIV were asked for resuscitator counseling, whose opinion determined the possibility of an ICU transfer or not. Sociodemographic and health characteristics were presented as numbers, mean, and standard deviations (SDs) values, and percentages. Differences in sociodemographic and health characteristics between who died and who survived were analyzed using t-test for differences in means for independent-sample and chi-square test for proportions.

## Results

Mean age of this hospital-based sample was 69.1 ± 14.5 years, men were largely more represented than women (73.6% vs. 26.4%), and both gender groups showed a higher percentage of patients aged > 70 years compared to patients aged < 50 years (41.3% vs. 9.3%). Main comorbidity was hypertension (64.5%) followed by cardiovascular disease (54.5%), chronic kidney disease (47.45%), and diabetes mellitus type 2 (30.5%). We subdivided the patients in two groups according to the admission date (before and after March 29, 2020). The first group (T1) was characterized by our relative limited knowledge about clinical features and adverse health-related outcomes of COVID-19, while for the second group (T2), our skill in the clinical management of these patients improved, with a more strategic organization of clinical and human resources.

Table 1 shows sociodemographic and clinical characteristics, therapeutic approaches and clinical outcomes of these two groups. The number of patients, age, and the gender distribution were similar in the two groups. At admission, both groups had the same illness severity: T1 group had a mean arterial oxygen partial pressure to fractional inspired oxygen) of 192 ± 77.2 vs. 198.1 ± 86 of the T2 group. Similarly, there is no statistically significant difference between the two groups about comorbidities. A different therapeutic approach was also evident in the two groups. In the T2 group, most of patients (56.9%) were treated with the therapeutic dose of enoxaparin compared with only 43.1% of patients in the T1 group (p < 0.19). For the T2 group, we used less lopinavir-ritonavir (2.1% vs. 96.9%, p < 0.01), more dexamethasone (73.9% vs. 26.1%, p < 0.01), and more hydroxychloroquine (92.1% vs. 78.9%, p = 0.15) compared with the T1 group. About non-invasive respiratory supports, continuous positive airway pressure was used much more while bilevel positive airway pressure was used less in T2 group (65.0% and 19.2%, respectively) than in T1 group (36.0% and 37.8%, respectively) (p < 0.05). Higher percentages of COVID-19 patients were transferred from T1 group in ICU (32.6% vs. 22%), without statistically significant difference respect to T2 group, but we observed a statistically significant lower mortality in the T2 group compared with the T1 group (17.1% vs. 52.2%, p < 0.01) (Table 1). More patients from T1 group died in our intermediate RICU, but without statistically significant difference compared with the T2 group (19.6% vs. 9.76% p < 0.33). Finally, we observed a significantly difference in terms of mortality among the patients transferred in ICU from intermediate RICU (100% in T1 group vs. 33.3% in T2 group, p < 005) and the average length of stay in intermediate RICU of ICU-transferred patients who survived in T1 and T2 was significantly longer than those who died (who died 3.3 ± 2.8 days vs. who survived 6.4 ± 3.3 days, p < 0.05). The average length of stay in ICU of patients who were transferred from intermediate RICU but died was 12.2 ± 7.6 days. Table 2 shows differences in the setup of intermediate RICU between T1 and T2.

**Table 1:** Sociodemographic and clinical characteristics, therapeutic approaches and clinical outcomes of COVID-19 patients hospitalized in an intermediate Respiratory Intensive Care Unit (RICU) subdivided in two groups T1 and T2 according to the admission date (before and after March 29, 2020)

	T1 group	T2 group	P = / <
N	46 patients	41 patients	
Sex (women/men)	12/34 (73.9% men)	11/30 (73.2% men)	0.87**
Age (years)	69.8 ± DS 13.5	68.4 ± DS 15.8	0.66*
Patients with medical chronic conditions (> 1)	45.3%	54.7%	0.51**
Therapy			
Lopinavir-ritonavir	96.9%	2.1%	< 0.01**
Dexamethasone	26.1%	73.9%	< 0.01**
Azithromycin	83.3%	90.6%	0.49**
Hydroxychlorochine	78.9%	92.1%	0.15**
NAC	77.7%	70.96%	0.64**
Tocilizumab	9.5%	3.12%	0.44**
Enoxaparin	43.1%	56.9%	0.19**
PaO_2_/FiO_2_ ratio	192 ± 77.2	198 ± 86.9	0.73*
Non-invasive respiratory support at admission			
HFNC	26.2%	15.8%	0.021**/***
CPAP	36.0%	65.0%	
BPAP	37.8%	19.2%	
Clinical outcomes			
Transferred to ICU	32.6%	22%	0.39**
Dead in ICU	100%	33.3%	0.005**
Dead in the intermediate RICU	19.6%	9.76%	0.33**
Transferred to GW	43.48%	21.95%	0.06**
Still hospitalized in intermediate RICU	4.35%	46.34%	0.001**

Data are showed as mean ± standard deviation for continuous and as percentage (%) for categorical variables

NAC: N acetyl cysteine; Pa02/FiO2: arterial oxygen partial pressure to fractional inspired oxygen; HFNC: high-flow nasal cannula; CPAP: continuous positive airway pressure; BPAP: bilevel positive airway pressure; ICU: Intensive Care Unit; GW: general ward

^*^Student t test for independent data

^**^Pearson Chi squared test

^***^The only comparisons between CPAP and the other non-invasive respiratory supports and between BPAP and the other non-invasive respiratory supports across time were significantly different (Bonferroni Inequality adjustment p < 0.05)

**Table 2:** Differences in the setup of an intermediate Respiratory Intensive Care Unit (RICU) between T1 and T2 according to the admission date (before and after March 29, 2020)

Intermediate RICU at T1	Intermediate RICU at T2
*Old structural allocation and organization of the intermediate RICU:*	*New structural allocation and organization of the intermediate RICU:*
Allocation in a building not still completely dedicated to COVID-19 patients and far from other different COVID-19 wards	Reallocation to a new building exclusively devoted to COVID-19 patients
No easy connection with ICU	Easy and quick connection with ICU
Routes for step up and step down not safe	Safe routes for step up and step down
*Old human resource organization of the intermediate RICU:*	*New human resource organization of the intermediate RICU:*
Pneumologists coming from general respiratory disease wards	Multidisciplinary staff with pneumologists expert of lung failure, physiotherapists, and intensivists
Reduced number of doctors and nurses	Increased number of doctors and nurses
Any knowledge of the COVID-19 physiopathology and correct clinical-therapeutic approach	Depth and gained experiences in real life on COVID-19 physiopathology and clinical-therapeutic approaches

*ICU* intensive care unit

## Discussion

The findings of the present study obtained in a hospital-based sample suggested that an intermediate level of hospital care may have the potential to modify survival in COVID-19 patients. Several reasons could explain the improved major clinical outcomes in the T2 group, i.e., mortality and length of stay in intermediate RICU of ICU-transferred patients. First, we improved the allocation of our intermediate RICU (Table 2). For the T1 group, we were at the ground floor of a building not still completely dedicated to COVID-19 patients, far from the ICU allocated in another building, and with the routes used for patient repositioning not completely safe. For the T2 group, we occupied the first floor of a building completely dedicated to COVID-19 patients, with better and quickly connection with ICU [3] and major possibility to access to care in safe way, using the same working protocol also with ED. Furthermore, there was a best use of human resources, with several new pneumologists, nurses and health’s workers hired thanks to regional funds allocated for the emergency (1 medical doctor for 6 patients and 1 nurse for 3 patients, and a multidisciplinary staff with pneumologists expert of lung failure, physiotherapists, and intensivists). For the T2 group, we also improved the organization of our clinical practice thanks to the expertise accumulated during the first days. It could be also hypothesized that a greater length of stay and evaluation in our intermediate RICU with non-invasive respiratory supports could favor a wait-and-see clinical approach that in selected patients could avoid the transfer in ICU, where mortality is linked not only to COVID-18 infection and its complications.

Moreover, also our therapeutic approach changed in the T2 group and most of patients (56.9%) were treated with the therapeutic dose of enoxaparin compared with only 43.1% of patients in the T1 group [15]. For the T2 group, we used less lopinavir-ritonavir [16], more dexamethasone [17], and more hydroxychloroquine [18] compared with the T1 group. Therefore, in the T2 group, we preferred to use dexamethasone over lopinavir-ritonavir in consideration of favourable clinical outcomes reached in recent studies. The combination of lopinavir-ritonavir used in HIV therapy and prevention has been widely administered during the first phase of COVID-19 pandemic, especially in mild patients (Stage I) [9] and during the first days after hospital admission. In the first phase, when the viral replication plays a pivotal pathogenetic role, antiviral drugs could be crucial in limiting viral-induced organ damage. In previous retrospective single-center studies, lopinavir-ritonavir was utilized in 90% of the patients hospitalized in South Korea [19] and in 82% of the critical ill patients hospitalized in Italy [20], confirming how this anti-viral drug was widely used in clinical practice worldwide. Nevertheless, some recent observational studies and randomized clinical trials (RCTs) have raised some concerns about its usefulness. In fact, Cao and colleagues, in the first controlled, open-label trial of anti-viral therapy, showed that in patients with severe COVID-19, lopinavir-ritonavir administration was not associated with a statistically significant difference in the time to clinical improvement compared with the standard-care control group and the short-term mortality rates were not statistically significant different between the two groups [16]. One reason explaining these findings could be that the patients were enrolled during the pulmonary stage with hypoxia (Stage IIb) [9], when the viral pathogenicity may be only one lesser dominant aspect of the overall pathophysiology, and host inflammatory responses were the predominant pathophysiology. Moreover, a recent meta-analysis concluded that treatment with lopinavir-ritonavir had no significant benefit in modifying mortality and ARDS rates in COVID-19 patients, but, on subgroups analysis, the lopinavir-ritonavir group had a lower rate of ARDS, although this difference was not statistically significant [21]. However, it should also be remembered that numerous ongoing RCTs around the world are currently evaluating the efficacy of lopinavir-ritonavir in COVID-19 patients.

Regarding the use of corticosteroids in COVID-19, there was great uncertainty about its administration during the first part of the pandemic due to lack of reliable clinical studies demonstrating its efficacy. In COVID-19, corticosteroids have primary been thought about as a mean to stave off the “cytokine storm” and his consequences like ARDS. As this usually happens in the first 5–7 days, ideally, steroid therapy should be tried in this period, particularly at the onset of dyspnea or even earlier to prevent the progression of “cytokine storm”. However, most of the studies on the use of corticosteroids in COVID-19 have shown variable findings, principally because of marked heterogeneity in the methodology used with considerable variations in the timing of initiation of steroid treatment, type, and dosage of steroids. The principal corticosteroids used in most of these studies and other ongoing RCTs have been methylprednisolone and dexamethasone because of their high bioavailability in the lung. Theoretically, methylprednisolone has the advantage of parenteral administration, a quicker onset of action and a shorter duration of action compared to dexamethasone. However, the most robust data on corticosteroids in COVID-19 came from the RECOVERY trial on dexamethasone, the only controlled, open-label trial conducted on 2104 patients assigned to receive dexamethasone and 4321 to receive usual care, which showed the most significant 28-day mortality benefit with low dose dexamethasone (6 mg per day oral or intravenous for 10 days) [22]. The RECOVERY trial showed an impressive mortality reduction among the sickest COVID-19 patients on invasive mechanical ventilation and among patients in oxygen therapy (with or without mechanical ventilation). In addition, COVID-19 patients on dexamethasone had a statistically significant reduction of length of hospital stay and an earlier likelihood of discharge. No benefit was observed in mild-to-moderate COVID-19 patients requiring no oxygen. However, more studies are still necessary to substantiate conclusive benefit with corticosteroid use in COVID-19 [22].

As regard hydroxychloroquine in COVID-19 pandemic, this drug has been widely used to reduce inflammation. In fact, an in vitro antiviral effect has been demonstrated on SARS-Cov-2, with chloroquine concentration of 0.36 mg/L that decreased viral load by 50% in a cell model [23]. However, to date, the administration of hydroxychloroquine in COVID-19 patients has been decreased due to its side effects and uncertain clinical benefits and all ongoing clinical trials with hydroxychloroquine used different dosing regimens, resulting in various concentrations [24]. Recently, Kannan and colleagues proposed a multiscale non-invasive model (Quasi-3D-based numerical formulation) to predict gastric and intestinal damage secondary to the use of hydroxychloroquine; this tool could be helpful in predicting the drug toxicity and optimizing the dosing regimen of hydroxychloroquine in COVID-19 patients [25]. Moreover, since the use of functional respiratory tests has been evaluated as a potential vehicle of SARS-Cov-2 infection, Kannan and colleagues provided a computational spirometry basal techniques (Quasi-3D model) that could offer fast and reliable information about bronchial caliber and morphology with a non-invasive approach to predict the lung health in COVID-19 patients [26].

## Conclusions

The present findings suggested that some hospital units designed for monitoring and treating respiratory patients whose illness is at a level of severity that is intermediate between that which requires ICU facilities and that which can be managed on a conventional ward may have the potential to modify survival also in COVID-19 patients particularly in the present phase of a more skilled clinical management of the pandemic. However, further larger studies are still necessary to verify that the modified therapeutic approaches could have changed clinical outcomes in these patients.

## Abbreviations

SARS-CoV-2: Severe acute respiratory syndrome coronavirus 2
COVID-19: Coronavirus disease 2019
RICU: Respiratory Intensive Care Unit
ICU: Intensive Care Unit
NAC: N acetyl cysteine
Pa02/FiO2: Arterial oxygen partial pressure to fractional inspired oxygen
HFNC: High-flow nasal cannula
CPAP: Continuous positive airway pressure
BPAP: Bilevel positive airway pressure
GW: General ward
RCTs: Randomized clinical trials
